# One hundred twelve cases of 46, XY DSD patients after initial gender assignment: a short-term survey of gender role and gender dysphoria

**DOI:** 10.1186/s13023-021-02039-1

**Published:** 2021-10-09

**Authors:** Liping Hou, Ming Zhao, Lijun Fan, Bingyan Cao, Jiajia Chen, Yonghua Cui, Michel Polak, Chunxiu Gong

**Affiliations:** 1 Department of Endocrinology, Genetics and Metabolism, Beijing Children’s Hospital, Capital Medical University, National Center for Children’s Health, Beijing, 100045 China; 2 Department of Endocrinology, Shenzhen Children’s Hospital, Shenzhen, 518038 China; 3 Department of Psychiatry, Beijing Children’s Hospital, Capital Medical University, National Center for Children’s Health, Beijing, 100045 China; 4 Pediatric Endocrinology Diabetology and Gynaecology, Hôpital Universitaire Necker Enfants-Malades, AP-HP, Paris, France

**Keywords:** 46, XY disorders of sex development, Gender assignment, Gender role, Gender dysphoria

## Abstract

**Background:**

46, XY disorders of sex development (46, XY DSD) are congenital disorders with 46, XY chromosomal karyotype but inconsistent gonadal/phenotypic sex. One of the biggest concerns for parents and clinicians is the gender assignment. However, there is no standard uniform of care nor consensus at present. We sought to evaluate the current treatment's rationality and provide a reference basis for the gender reassignment in 46, XY DSD patients with a specific diagnosis.

**Methods:**

We conducted a cross-sectional survey of gender role with the Pre-school Activities Inventory (PSAI), the Children's Sex Role Inventory (CSRI) in 46, XY DSD patients and set up control groups comparison. Psychiatrist assessed gender dysphoria in patients ≥ 8-year-old with the criteria of diagnostic and statistical manual of mental disorders, 5th edition (DSM-5).

**Results:**

A total of 112 responders of 136 patients participated in this study (82.4%, aged 2–17.8 years, median age: 4-year-old). The follow-up period was from 6 months to 10 years (median: 2 years). Twenty-five females were reassigned to the male gender after a specific diagnosis (16/25 (64%) in 5 alfa-reductase-2 deficiency (5α-RD2), 5/25 (20%) in partial androgen insensitivity syndrome (PAIS), 4/25 (16%) in *NR5A1*gene mutation). Male gender assignment increased from 55.3 (n = 62) to 77.7% (n = 87). The median PSAI score was similar to the control males in *5α-RD2*, PAIS, and *NR5A1* gene mutation groups (*p* > 0.05); while identical to the control females in complete androgen insensitivity syndrome (CAIS) and *CYP17A1* gene mutation groups (*p* > 0.05). PSAI score of children raised as male was higher than those of CAIS and CYP17A1 groups raised as female (*p* < 0.05). CSRI scale showed no statistical differences in the consistency of gender roles and reassigned gender between 46, XY DSD patients and control groups (*p* > 0.05). None of the patients over 8-year-old (n = 44) had gender dysphoria.

**Conclusion:**

The reassigned gender in 46, XY DSD patients is consistent with their gender role during early childhood. None of them had gender dysphoria. The molecular diagnosis, gonadal function, and the gender reassignment are congruent within our Chinese cohort. Long-term follow-up and more evaluation are still required.

## Background

46, XY disorders of sex development (46, XY DSD) are congenital disorders with 46, XY chromosomal karyotype but inconsistent gonadal/phenotypic sex [1]. The newborn infant with ambiguous external genitalia is frequently described as a clinical emergent situation because that is distressing the parents [2]. Gender assignment, which is usually base on the appearance of external genitalia in newborns with typical genitalia at birth, becomes one of the most difficult decisions for clinicians when a newborn with an atypical genital. Historically, gender assignment consisted of early surgery to achieve cosmetically normal external genitalia and excise the gonads to match the assigned gender. However, in recent years, elective surgery has been deferred to allow the children to be part of the decision regarding gender assignment and surgical intervention [3–5]. Due to the high binarism of sex in China, the DSD child shames parents. Those DSD children undiagnosed and untreated in infancy or early childhood are often subjected to severe social discrimination. In addition, there is no other category than male or female in official documents (such as birth certificate, registered permanent residence, passport, public facilities), some 46, XY DSD patients have to face the problem of early gender assignment before being medically assessed and getting a social gender when registered by parents. It is a problem without experienced endocrinologists in China.

The management of gender assignment in 46, XY DSD, is individualized with the cooperation of multidisciplinary team (MDT), preserving gonadal function, avoiding irreversible surgery, keeping options open, and try to align gender identity to be compatible with the assigned gender and avoid gender dysphoria (GD) [6]. The team of the department of endocrinology, genetics and metabolism of Beijing Children's Hospital, Capital Medical University, has accumulated a large cohort of 46 XY DSD patients in the past decade. With genetics and gene detection available in recent years, part of our DSD children was diagnosed with a definite genetic diagnosis, making gender assignment, outcome prediction, genetic counselling, and higher life quality possible. Under the cooperation of the MDT, we discussed the gender reassignment or endorsement with their parents according to the analysis of genetics, androgenic level, internal and external genital anatomy, and parental view. Finally, the decision rests with the parental consent. If the child is raised as female, we propose to leave intact the gonads until puberty when she could confirm her gender. If the child is raised as male, we suggested hypospadias repairmen operation at an appropriate time [7] to help parents and patients cope with the social pressure associated with atypical genitalia.

At present, only a few relevant works conducted preliminary discussions on the gender assignment of 46, XY DSD patients in China, and none of them have been involved in gender dysphoria neither genetic diagnosis for all involved in the research cases. In this study, we conducted a short-term survey of gender role (GR) and assessed the existence of GD in 46, XY DSD to evaluate the treatment's rationality and provide a reference basis for gender reassignment. The role of the children will need to be refined further in our practice.

## Materials and methods

### Participants

The Ethics Committee of Beijing Children's Hospital affiliated with Capital Medical University approved this study. We reviewed 46, XY DSD patients' data and conducted a cross-sectional survey from January 2019 to December 2020. The following were the inclusion criteria: ① Atypical external genitalia (exhibited as female-typical external genitalia, small penis, hypospadias of all degrees with or without cryptorchidism). ② 46, XY Chromosome karyotype; ③ Imaging or pathological results suggesting or showing that the gonadal tissue contains testis, respectively; ④ Genetic test results to support the diagnosis of 46, XY DSD; ⑤ All the participants were over two-year-old and followed up at least six months after the gender reassignment. The exclusion criteria were: ① Intellectual disability. ② Imaging or pathological results show the gonadal tissue contains ovaries or ovarian testis tissue. ③ Incomplete data collection or informed consent.

### Groups

Patients were divided into five groups based on genetic diagnosis, all the patients had pathogenic variants: ① 5 alfa-reductase-2 deficiency(*5α-RD2*); ② Complete androgen insensitivity syndrome (CAIS, female-typical external genitalia, clitoris length < 1 cm); ③ Partial androgen insensitivity syndrome (PAIS, clitoris length ≥ 1); ④ *NR5A1* gene mutation; ⑤ *CYP17A1* gene mutation. Control groups consist of unaffected, age-matched males and females who came from our Children’s healthcare department.

### Methods

**Fig. 1:**
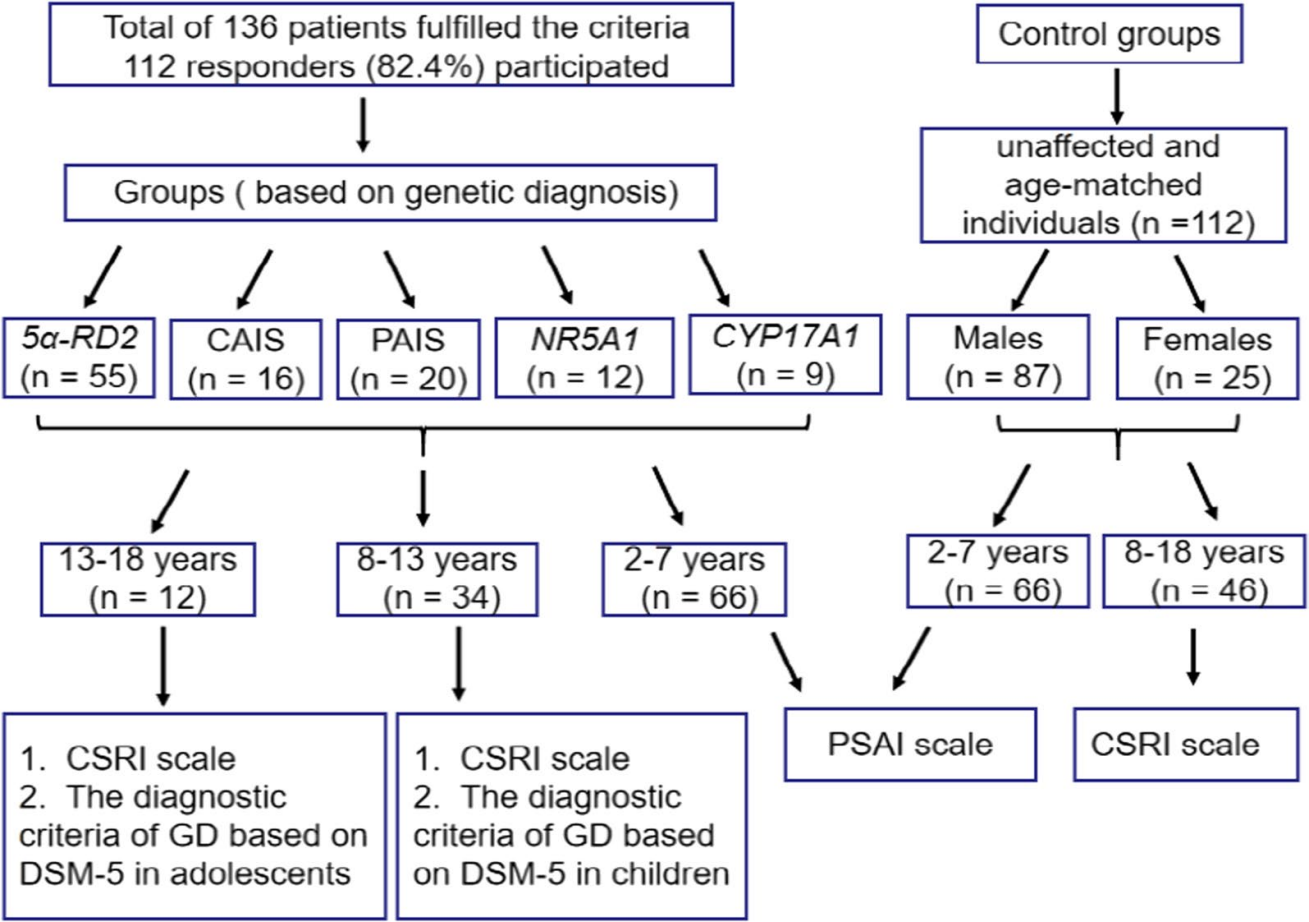
Flowchart of grouping and follow-up. *5α-RD2*, 5 alfa-reductase-2 deficiency; CAIS, complete androgen insensitivity syndrome; PAIS, partial androgen insensitivity syndrome; *NR5A1*, *NR5AI* gene mutation; *CYP17A1*, *CYP17A1* gene mutation; PSAI, Pre-school Activities Inventory; CSRI, The Children's Sex Role Inventory; GD, gender dysphoria; DSM-5, diagnostic and statistical manual of mental disorders, 5th edition

All the questionnaire surveys were conducted during the outpatient visit. The Pre-school Activities Inventory (PSAI) scale and The Children's Sex Role Inventory (CSRI) scale was used to assessed GR for patients aged 2–7 years and 8–18 years, respectively. Psychiatrist assessed GD in children and adolescents ≥ 8-year-old with the criteria of DSM-5. Figure 1 shows the flowchart.

### The evaluation and judgment of Gender role and gender dysphoria

(1) **The Pre-school Activities Inventory (PSAI) **[8]: The reliability of the PSAI scale was determined acceptable by the Cronbach’s alpha coefficients of 0.674. It is a 24-item standardized psychometric parent-report instrument for 2–7 years old children and has good reliability and validity in Chinese culture. It consists of 7 toys, 11 activities, six personality traits. Each item scores on a 5-point Likert scale (1 = never, 2 = rarely, 3 = sometimes, 4 = often, and 5 = very frequent). Masculine and feminine content each account for half (total score of 60 points). PSAI score = (masculine score − feminine score) × 1.1 + 48.25. The higher the PSAI score obtained, the higher the degree of masculinity.

(2) **The Children's Sex Role Inventory (CSRI) **[9]: The reliability of the CSRI scale was determined acceptable by the Cronbach’s alpha coefficients of 0.83 and 0.77 for the masculinity and the femininity subscales, respectively. It is a 52-item standardized psychometric self-report instrument used for over 8-year-old children that consists of 17 masculine options, 15 feminine options, and 20 neutral options. Each item bases on very conforming, most conforming, partially conforming, non-compliant options and the corresponding scores are 4-1 points. Masculine score = total masculine score/17; feminine score = total feminine score/15, CSRI score = masculine score − feminine score, positive value (CSRI score > 0) indicates masculine tendency, negative value (CSRI Score < 0) indicates feminine tendency.

(3) **The diagnosis criteria of GD based on DSM-5 in children **[10]: It is used for assessing the existence of GD in patients of 8–13 years old. It comprises eight indicators. When manifested more than six indicators and at least six months duration can diagnose as GD (A1 is necessary, that is, "I strongly hope to be another gender or insist that one gender is the other gender or some alternative gender different from the assigned gender"). Three of them (A1, A7, and A8) refer to the child's desire or dislike, and the other five (A2–A6) are behaviors and preferences that can be readily observed. The diagnosis of GD was appraised by a psychiatrist based on a clinical interview.

(4) **The diagnosis criteria of GD based on DSM-5 in adolescents **[10]: It is used for assessing the existence of GD in patients ≥13-year-old. It consists of six indicators. When manifested more than two indicators and at least six months duration can diagnose as GD. The diagnosis of GD was appraised by a psychiatrist based on a clinical interview.

### Statistical processing

Statistical analysis was carried out by statistical software SPSS 20. All the data did not conform to the normal distribution and were described as M(QL–QU) and percentage (%). The Kruskal–Wallis H test compared the PSAI score of different etiological groups. The Bonferroni-corrected Mann–Whitney U test compared pairwise comparisons. A Chi-square test was used to compare the CSRI score between patients and control groups. Fisher's exact probability method was used when the theoretical minimum frequency is less than 1. *p* < 0.05 was considered statistically significant.

## Results

1. **Participants and clinical manifestation:** A total of 136 patients fulfilled the inclusion and exclusion criteria, 112 (82.4%) responders aged 2–17.8 years old (median: 4-year-old) took part in this investigation, including patients of 2–7 years old (n = 66) and ≥ 8-year-old (n = 46). The External Masculinization Score (EMS) is used to evaluate the degree of masculinization [11]. The clinical manifestations of 46, XY DSD patients with different ages and etiologies is shown in Table 1.

**Table 1:** The clinical manifestations of 46, XY DSD patients with different ages and etiologies

Age	Group	n	Clinical manifestation				EMS M (QL–QU)	HCG—well-responsive n (%)	Androgen therapy # n (%)	Puberty n (%)
			Female genital with Labia mass or Cryptorchidis n (%)	*One phenotype n (%)	**Two phenotypes n (%)	***Three phenotypes n (%)				
2–7 years (n = 66)	*5α-RD2*	33	7 (21.2%)	4 (12.1%)	20 (60.6%)	2 (6.1%)	6.0 (3.0–7.0)	33 (100%)	33 (100%)	–
	CAIS	8	8 (100%)	0	0	0	2.0 (0.3–2.0)	–	–	–
	PAIS	12	1 (8.3%)	1 (8.3%)	8 (66.7%)	2 (16.7%)	4.5 (3.0–6.4)	12 (100%)	12 (100%)	–
	*NR5A1*	7	1 (14.3%)	0	5 (71.4%)	1 (14.3%)	6.0 (3.0–7.0)	7 (100%)	7 (100%)	–
	*CYP17A1*	6	6 (100%)	0	0	0	2.0 (2.0–2.3)	0	0	–
8–18 years (n = 46)	*5α-RD2*	22	5 (22.7%)	0	12 (54.5%)	5 (22.7%)	5.5 (3.0–7.0)	22 (100%)	22 (100%)	7 (31.8%)
	CAIS	8	8 (100%)	0	0	0	2.0 (2.0–2.9)	–	–	–
	PAIS	8	3 (37.5%)	0	5 (62.5%)	0	5.0 (3.0–7.0)	8 (100%)	7 (87.5%)	3 (37.5%)
	*NR5A1*	5	0	0	2 (40%)	3 (60%)	6.0 (5.5–7.2)	5 (100%)	5 (100%)	2 (40.0%)
	*CYP17A1*	3	2 (66.7%)	0	1 (33.3%)	0	2.0 (–)	1 (33.3%)	1 (33.3%)	1 (33.3%)

*5α-RD2*, 5 alfa-reductase-2 deficiency; CAIS, complete androgen insensitivity syndrome; PAIS, partial androgen insensitivity syndrome; *NR5A1*, *NR5AI* gene mutation; *CYP17A1*, *CYP17A1* gene mutation; HCG, Human Chorionic Gonadotropin; EMS, External Masculinisation Score, the lower the score is, the higher degree of under-masculinization. ^#^Androgen therapy administered for only 3 months before hypospadias repairmen operation.*Micro-penis or Hypospadias or Cryptorchidis;**Any two combinations of Micro-penis, Hypospadias, Cryptorchidis. ***Micro-penis, Hypospadias and Cryptorchidis

2. **Gender reassignment:** All of the patients were given parental consent for gender reassignment. Twenty-five females were reassigned to the male gender after a specific diagnosis (16/25 (64%) in 5α-RD2, 5/25 (20%) in PAIS, 4/25 (16%) in *NR5A1*gene mutation). Male gender assignment increased from 55.3 (n = 62) to 77.7% (n = 87). The age of gender reassignment was 0.2–14.6 years old (median: 2-year-old). The follow-up period was 6 months to 10 years (median: 2 years). The age of control groups (87 males, 25 females) ranges from 2.4 to 16.8 years old, with a median age of 4.7 years. There was no statistically differences in age between 46, XY DSD patients and control groups (all *p* > 0.05) (Table 2).

**Table 2:** The gender reassignment in 46, XY DSD patients with different ages and etiologies

Age	Group	n	Age of first vist (year) M (QL–QU)	Age of gender re-assignment (year) M (QL–QU)	Follow-up period (year) M (QL–QU)	Age at study (year) M (QL–QU)	Male gender at birth n (%)	Male gender after diagnosis n (%)
2–7 years (n = 66)	*5α-RD2*	33	1.5 (0.8–2.7)	2.0 (1.1–3.1)	2.0 (1.4–2.5)	4.0 (3.5–5.6)	25 (75.7%)	33 (100%)
	CAIS	8	2.6 (1.9–4.6)	2.9 (2.0–4.6)	0.9 (0.5–1.2)	4.1 (3.0–5.3)	0	0
	PAIS	12	1.2 (0.7–2.1)	1.6 (0.8–2.4)	2.0 (1.0–3.2)	4.0 (2.5–5.1)	11 (91.6%)	12 (100%)
	*NR5A1*	7	1.0 (0.6–1.3)	1.3 (1.0–1.5)	2.0 (1.0–2.0)	3.0 (2.5–3.2)	5 (71.4%)	7 (100%)
	*CYP17A1*	6	1.9 (1.2–3.2)	2.1 (1.3–4.1)	2.6 (1.3–4.0)	5.9 (4.0–6.7)	0	0
	Control Males	52	–	–	–	4.7 (4.0–5.1)^a^	–	–
	Control Females	14	–	–	–	5.0 (3.3–6.2)^b^	–	–
8–18 years (n = 46)	*5α-RD2*	22	5.8 (1.0–9.7)	6.7 (1.3–11.2)	4.3 (1.1–7.8)	10.6 (8.9–13.0)	14 (63.6%)	22 (100%)
	CAIS	8	7.2 (1.5–8.9)	7.9 (6.9–8.9)	1.0 (0.7–3.1)	9.5 (8.6–10.0)	0	0
	PAIS	8	4.4 (2.2–6.3)	4.6 (3.0–11.1)	4.5 (1.7–6.3)	10.5 (8.0–13.7)	3 (37.5%)	7 (87.5%)
	*NR5A1*	5	6.1 (1.1–12.4)	6.3 (1.9–12.9)	1.9 (1.4–7.3)	10.0 (8.1–14.3)	3 (60.0%)	5 (100%)
	*CYP17A1*	3	12.0 (–)	12.7 (–)	1.9 (–)	13.2 (–)	1 (33.3%)	1 (33.3%)
	Control Males	35	–	–	–	10.4(9.5–12.0)^c^	–	–
	Control Females	11	–	–	–	10.5(9.7–12.7)^d^	–	–

*5α-RD2*, 5 alfa-reductase-2 deficiency; CAIS, Complete androgen insensitivity syndrome; PAIS, Partial androgen insensitivity syndrome; *NR5A1*, *NR5AI* gene mutation; *CYP17A1*, *CYP17A1* gene mutation; M, Median; QL, 25%; QU, 75%; Mann–Whitney U test was used to compare the age at study in 46, XY DSD patients and control groups. ^a^Z score =  − 1.849, *p* = 0.065 > 0.05; ^b^Z score =  − 0.115, *p* = 0.919 > 0.05; ^c^Z score =  − 0.435, *p* = 0.668 > 0.05; ^d^Z score =  − 0.789, *p* = 0.447 > 0.05

## The consistency between gender role and the assigned gender:

**Fig. 2:**
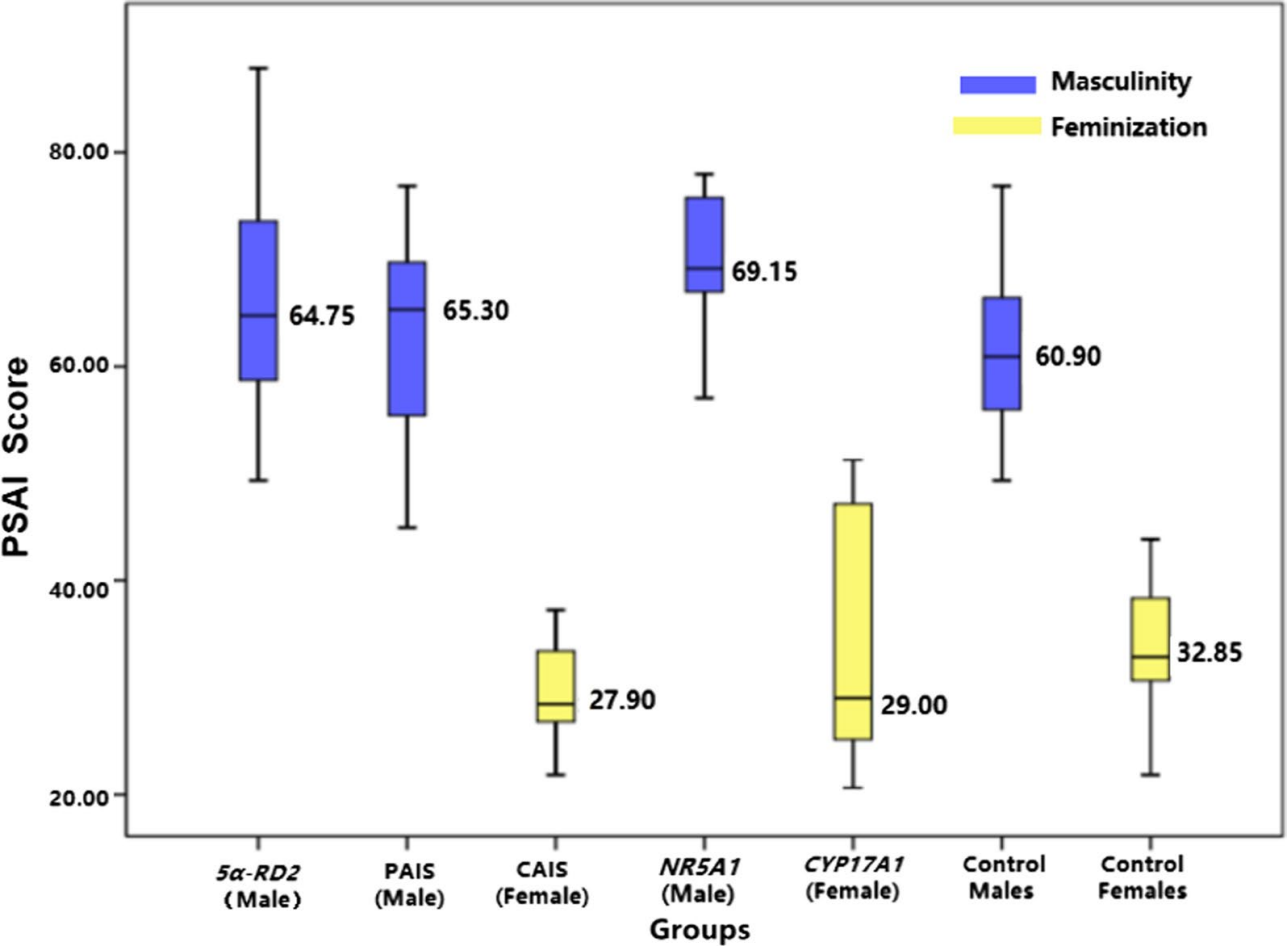
The comparison of PSAI score of 46, XY DSD children aged 2–7 years with different etiologies. *5α-RD2*, 5 alfa-reductase-2 deficiency; CAIS, complete androgen insensitivity syndrome; PAIS, partial androgen insensitivity syndrome; *NR5A1*, *NR5AI* gene mutation; *CYP17A1*, *CYP17A1* gene mutation; PSAI, pre-school Activities Inventory. The higher the PSAI score obtained, the higher the degree of masculinity

(1) **PSAI score** 66 cases of patients and control groups (52 males and 14 females) aged 2–7 years old completed the PSAI scale. The results showed that the median PSAI score of *5α-RD2* deficiency, PAIS, and *NR5A1* gene mutation group (median PSAI score were 64.75, 65.30, 69.15, respectively) was similar to control males (median PSAI score was 60.90, all *p* > 0.05). They had typically masculine gender roles and consistent with their reassigned gender (100%). PSAI score of CAIS (median PSAI score was 27.90) and *CYP17A1* gene mutation group (median PSAI score was 29.00) were similar to control females (median PSAI score was 32.85, all *p* > 0.05). They had typically feminine gender roles and consistent with their reassigned gender (100%, 83.3%, respectively, set out in Table 3). The PSAI score of patients with *5α-RD2* deficiency, PAIS, and *NR5A1* gene mutation (raised as male) was higher than those of patients with CAIS and *CYP17A1* gene mutation group (raised as female) (all *p* < 0.05, Table 3 and Fig. 2).

**Table 3:** The results of PSAI in 46, XY DSD children with different etiologies (2–7 years)

PSAI Score (24 items)	Groups (n = 66)							
	*5α-RD2* (M) n = 33	PAIS (M) n = 12	*NR5A1* (M) n = 7	CAIS (F) n = 8	*CYP17A1* (F) n = 6	Control (M) n = 52	Control (F) n = 14	
Masculine score (QL–QU)	38.00 (36.8–45.0)	37.0 (34.3–42.8)	46.0 (44.0–50.0)	27.0 (22.5–31.5)	29.5 (23.0–37.8)	37.5 (34.0–41.0)	30.0 (24.0–31.5)	
Feminine score (QL–QU)	24.00 (21.0–28.0)	25.0 (21.3–28.0)	26.0 (19.0–29.0)	43.0 (39.0–50.0)	46.0 (41.0–47.3)	26.5 (23.0–29.0)	40.5 (35.5–47.3)	
PSAI score (QL–QU)	64.75 (58.7–73.6)	65.30 (55.1–70.5)	69.15 (65.8–77.9)	27.90 (26.5–34.8)	29.00 (24.0–48.2)	60.90 (55.9–66.7)	32.85 (30.6–38.4)	
Consistency n (%)	33 (100%)	12 (100%)	7 (100%)	8 (100%)	5 (83.3%) *	52 (100%)	13 (100%)	

Compared to		Comparison of PSAI score (*p* value) **						
*5α-RD2* (M) n=33	--	1.000	1.000	0.000	0.000	1.000	0.000	
PAIS (M) n=12	--	–	1.000	0.001	0.010	1.000	0.000	
*NR5A1* (M) n=7	--	–	–	0.000	0.000	0.936	0.000	
CAIS (F) n=8	--	–	–	–	1.000	0.000	1.000	
*CYP17A1* (F) n=6	--	–	–	–	–	0.007	1.000	
Control (M) n=52	--	–	–	–	–	–	0.000	

M, male; F, female; *5α-RD2*, 5 alfa-reductase-2 deficiency; CAIS, complete androgen insensitivity syndrome; PAIS, partial androgen insensitivity syndrome; *NR5A1*, *NR5AI* gene mutation; *CYP17A1*, *CYP17A1* gene mutation; PSAI, pre-school Activities Inventory. PSAI score = (masculine score—feminine score) × 1.1 + 48.25. The higher the PSAI score obtained, the higher the degree of masculinity

*The Chi-Square test (Fisher exact probability method) was used to compare the consistency of the gender role and assigned gender between CYP17A1 gene mutation group (assigned as female) and control females, the result showed there was no statistical difference between them (*p* = 0.30 > 0.05)

**The Kruskal–Wallis H test was used to compare the PSAI score between different etiologies groups and control groups. There was statistical difference between them (Chi-Square = 71.168, *p* < 0.001).The Mann–Whitney adjusted by Bonferroni Pairwise comparisons were performed by U-test: the PSAI score of patients with 5α-RD2 deficiency, PAIS, and NR5A1 gene mutation (assigned as male) were higher than those with CAIS and *CYP17A1* gene mutation (assigned as female) (*p* < 0.05). The PSAI score of patients with CAIS and CYP17A1 gene mutation were similar to the control females (all *p* > 0.05)

(2) **CSRI score** 46 patients and control groups (35 males and 11 females) aged 8–18 years old completed the CSRI scale. A positive value of the CSRI score indicates masculinity, while a negative value indicates feminization. The CSRI score indicated masculinity were: 9/22 (40.9%) in *5α-RD2* deficiency, 5/7 (71.4%) in PAIS, 3/5 (60%) in *NR5A1*gene mutation, 4/8 (50%) in CAIS, 18/35 (51.4%) in control males and 1/11 (9.1%) in control females. Compared with the control males, there are no statistical differences in the consistency of gender role and reassigned gender in *5α-RD2* deficiency, PAIS and *NR5A1* gene mutation (*p* = 0.587, 0.428, 1.000, respectively, all *p* > 0.05). Compared with the control females, there are no statistical difference in the consistency of gender roles and reassigned gender in CAIS (*p* = 0.111 > 0.05) (Table 4).

**Table 4:** THE comparison of CSRI score of 46, XY DSD patients with different etiologies (8–18 years)

Groups	Gender assignment n = 46	CSRI score (52 items)			Consistency *	
		Masculine score M (QL–QU)	Feminine score M (QL–QU)	CSRI score M (QL–QU)	n (%)	*p* value
*5α-RD2*	Male (n = 22)	2.83 (2.65–3.18)	3.11 (2.67–3.32)	− 0.09 (− 0.45 to 0.32)	9 (40.9%)	0.587**
PAIS	Male (n = 7)	3.00 (2.89–3.11)	2.71 (2.37–2.95)	0.29 (− 0.05 to 0.36)	5 (71.4%)	0.428**
	Female (n = 1)	2.70 (–)	2.73 (–)	− 0.03 (–)	1 (100%)	–
CAIS	Female (n = 8)	2.58 (2.09–2.91)	2.86 (2.70–3.23)	− 0.28 (− 0.92 to 0.29)	4 (50%)	0.111***
*NR5A1*	Male (n = 5)	3.06 (2.51–3.19)	3.00 (2.63–3.11)	0.102 (− 0.12 to 0.22)	3 (60%)	1.000**
*CYP17A1*	Male (n = 1)	2.90 (–)	2.00 (–)	0.90 (–)	1 (100%)	–
	Female (n = 2)	3.15 (–)	3.60 (–)	− 0.45 (–)	2 (100%)	–
Control groups	Male (n = 35)	2.82 (2.47–3.06)	2.73 (2.40–3.00)	0.01 (− 0.20 to 0.30)	18 (51.4%)	–
	Female (n = 11)	2.23 (2.00–2.59)	2.70 (2.35–3.47)	− 0.42 (− 0.69 to − 0.34)	10 (90.9%)	–

*5α-RD2*, 5 alfa-reductase-2 deficiency; CAIS, complete androgen insensitivity syndrome; PAIS, partial androgen insensitivity syndrome; *NR5A1*, *NR5AI* gene mutation; *CYP17A1*, *CYP17A1* gene mutation; CSRI, The Children's Sex Role Inventory; CSRI score = masculine score–feminine score, positive value (CSRI score > 0) indicates masculinity, negative value (CSRI score < 0) indicates feminization

^*^Consistency of gender role and assigned gender

^**^The chi-square test (Fisher exact probability method) was used to compare the consistency of gender role and assigned gender between *5α-RD2* deficiency, PAIS, *NR5A1* and control males respectively, all *p* > 0.05

***The chi-square test (Fisher exact probability method) was used to compare the consistency of gender role and assigned gender between CAIS and control females (*p* = 0.111 > 0.05)

(3) **Gender dysphoria** The psychiatrist conducted a clinical interview with patients aged 8–13 years old (n = 34) and 13–18 years old (n = 12); none of them satisfies the diagnosis criteria of GD based on DSM-5.

## Discussion

This study evaluated our clinical management outcome by conducting a cross-sectional survey on the gender role and gender dysphoria in 112 cases of 46, XY DSD patients with a specific diagnosis after initial gender assignment. The results showed that the gender roles of all 46, XY DSD patients were consistent with their reassigned gender. There were no indications of gender dysphoria for the time being, and the reassigned gender was congruent in short-term results with our practice at the time when the cohort was taken care of. That is similar to the conclusion of a perspective from Brazilian, where precocious binary sex assignment and early surgery were judged by that team the elective is better management methods for DSD patients [12]. There is no convincing evidence in this cohort that this will lead to harmful catastrophic consequences in adulthood. We emphasize that our study reflects the practice in a specific context, a specific time, and a specific geographical area with the role of children in the decision process still to be defined. Moreover, gender reassignment, the focus of our work here, is not necessarily linked to the way of taking medical and surgical care of the child with a sex developmental variation.

Indeed, psychosexual development is complex and affected by multiple factors such as exposure to androgens, sex chromosome genes, social circumstances, and family dynamics [1]. It is conceptualized into three components: gender role, gender identity and sexual orientation. One of the most important parts of psychosexual development is gender identity, which refers to a person's internal sense of self as male, female, or the other sex [13]. Genetics, hormonal, neuroanatomical, and social factors are now considered major factors in forming a person's gender identity [14]. Although gender identity is difficult to predict, in most patients with DSD, the sex of rearing, which parents raise their children according to the sex that doctors assigned, and socialization is still the best predictor of long-term gender identity outcomes [15–17]. Gender role is defined as characteristics designated as masculine or feminine in personality, appearance, and behavior in a specific cultural and historical period (i.e., more typically masculine or feminine social roles). For most children, their gender role expression, behavior, interests, and preferences are linked to their experience as males or females (Gender identity). The gender role behavior of a person reflects the degree of gender identity to a certain extent. Those who exhibit continually atypical gender role behavior that differs from their assigned gender are more likely to develop GD [18]. Of note that the causal mechanism of GD may be unknown, but the importance of biological influences via genes and hormones is evident [19]. To reduce the risk of gender dysphoria or even gender reassignment in the future, we gave an appropriate recommendation for gender based on etiology diagnosis, gonadal function, and parental view. Finally, in *5α-RD2* deficiency, PAIS, *NR5A1* gene mutation, and those with HCG-well-responsive were recommended male gender. In contrast, all CAIS and those with HCG-poor-responsive of *CYP17A1* gene mutation were recommended female gender. The recommendation of male gender in our study is increased from 55.3 to 77.7%, which is similar to the research from Kreukels and Kolesinska [20, 21]. To understand whether there is any discrepancy between gender roles and the reassigned gender, we utilized the PSAI, which Chinese scholars had verified, to assess gender roles in 2–7 years old patients. The results revealed that children with 5α-RD2, PAIS, and *NR5A1 *gene mutation who were raised as males all have typical masculine gender roles, their PSAI scores are similar to those of the control males'. Children with CAIS and *CYP17A1* gene mutation are raised as females who have typical feminine gender roles. Their PSAI score is identical to those of control females'. Our data indicate that the gender role of these research group children with 46, XY DSD is consistent with their reassigned gender. The results are congruent with our practice of gender reassignment in this cohort.

It has been shown that prenatal androgens play a major role in developing sex differences in children's play behaviour [22]. Furthermore, to understand the effect of prenatal androgen on children's gender roles, we compared the PSAI score between different etiologies. We found that: PSAI score of children with 5α-RD2, PAIS, and *NR5A1* gene mutation who were raised as males were higher than those in the CAIS and *CYP17A1* gene mutation who were raised as females. That means male individuals with HCG-well-responsive exposed to androgen showed masculine behaviors while female individuals (patients of *CYP17A1* gene mutation with HCG-poor-responsive and CAIS) showed typical feminine behaviors and preferences. Our findings are similar to most previous studies [23–27]. Besides, Loch Batista [28] recently conducted a sexual, psychological evaluation of 144 patients with 46, XY DSD from 18 to 60 years old. They grouped the patients according to prenatal androgen exposure for specific DSD aetiology and found that prenatal androgen exposure has significant gender role and gender identity differences. Normal prenatal androgen exposure is associated with higher masculine psychological results. Combined with the results that literature as mentioned above, our data have also reinforced that the gender role of 46, XY DSD patients in childhood is affected by prenatal androgen exposure.

Since sexual psychological development develops over time, any assessment tool that measures gender role needs to be validated in the developmental spectrum of children, adolescents, and adults. It is inappropriate to apply the PSAI scale for patients of all ages. Although Khorashad [27] used the PSAI scale to investigate 16 male identifiers of 5α-RD2 deficiency aged 5–32 years old and also showed androgenic hormones played a role in the development of typical childhood gaming behavior, the reliability of the results would likely be affected by the possibility of recall bias due to the recall method. Besides, the gender type of self-perception may become more important than the gender type of self-observation as a child grows up [29]. Therefore, it is necessary to select a corresponding age scale to carry out the investigation. In this regard, we apply the CSRI, which Chinese scholars had verified, to conduct a gender role consistency survey on patients and control groups aged 8–18 years. The results showed that nearly half of the males score higher on the femininity scale, resulting the rates of consistency between CSRI scores and reassigned gender are low in 5α-RD2 and control males. However, there are no statistical differences in the consistency of gender roles and reassigned gender between DSD subsets and control groups. The reason why CSRI score showed femininity in these male gender is probably because Chinese collectivist culture emphasizes harmonious relationships in the collective. The expressive characteristics of feminine traits are usually conducive to establishing cooperative relationships, making males and females score higher on the femininity scale [9].

Parents and healthcare may regard gender-role behavior that is inconsistent with the assigned gender as an indication of gender dysphoria and question the decision of gender assignment might have been incorrect. However, literature studies show that gender roles are not always associated with gender identity or biological gender characteristics [30] and should not be used as an indicator of gender reassignment [1]. We also believe that gender role is only a reference to one of many dimensions. It is more important to know whether these children have GD or not, which can provide a reference basis for judging whether the recommended gender is correct, as well as to detect those children with signs of GD as early as possible and reevaluate as soon as possible. GD, previously known as gender identity disorder, is a significant inconsistency between the gender a person experiences or expresses and the assigned gender of psychological discomfort for at least 6 months [10]. Due to the disidentification of their sex at birth or assigned gender, children who experience gender dysphoria exhibit extreme and persistent gender incongruence or aberrant gender role behaviors, preferences, and interests. It represents a dimensional phenomenon that can occur with varying degrees of intensity, with the most extreme form accompanying the desire for social or physical transformation so that the body is as aligned with gender identity as possible [10, 31, 32]. DSD individuals with indistinguishable genitals have been declared to have a higher prevalence of GD than the general population [33], and they may experience clinically significant suffering that requires clinical attention. Thus, We referred these 46, XY DSD patients aged ≥ 8 years to a psychiatrist to assess GD according to the diagnostic criteria of gender dysphoria in children and adolescents described in DSM-5. The results revealed that: none of the patients met the diagnostic criteria of GD, and both patients and their parents were satisfied with their current gender. As the diagnosis of DSD in the past mainly relied on clinical and endocrine examinations, the aetiology of some children was not diagnosed and given the wrong gender assignment, leading to the reports that 63–66% of the children with 5α-RD2 deficiency raised as females changed gender to males in adolescence [34]. To minimize the occurrence of GD, we recommended assigning children with normal androgen exposure to males and those with weak or lack of androgen imprinting effects to females for children with a specific diagnosis. In combination with our findings, we believe that it is essential to reassign gender mainly based on molecular diagnosis and gonadal function. That corroborates a recent study [35], provides clinical gender assignment with another dimension of reference for psychological gender assignment and assists clinical decision-making. Further studies are needed to show that gender assignment is correlated with the medical and surgical care given, as it was not the present study's focus. A chapter of the importance of the multidisciplinary team to decide and continuous including psychological support to these patients all along adolescence.

## Conclusion

Gender assignment is always a complex, challenging and demanding experience in managing patients with DSD for clinicians. Our short-term observations reveal that the reassigned gender of 46, XY DSD patients is consistent with their gender roles and without any report of gender dysphoria. The gender reassignment and the molecular diagnosis and gonadal function are congruent within our Chinese cohort. However, factors impacting GD or gender identity are still unknown. We remain open to the continuous knowledge in that field that may alter the care of these patients. Long-term gender outcome of these children and further assessment is necessary. The role of the child in the process of care needs better defined, and more appropriate assessment tools are required to be developed in the future.

## Data Availability

The data are not publicly available due to privacy or ethical restrictions. The data that support the findings of this study are available on request from the corresponding author.

## Abbreviations

46, XY DSD: 46, XY disorders of sex development
MDT: Multidisciplinary team
PSAI: Pre-school Activities Inventory
CSRI: Children's Sex Role Inventory
DSM-5: Diagnostic and Statistical Manual of Mental Disorders, 5th edition
5α-RD2: 5 alfa-reductase-2 deficiency
PAIS: Partial androgen insensitivity syndrome
CAIS: Complete androgen insensitivity syndrome
NR5A1: Nuclear Receptor Subfamily 5 Group A Member 1
CYP17A1: Cytochrome P450 Family 17 Subfamily A Member 1
GR: Gender role
GD: Gender dysphoria
HCG: Human Chorionic Gonadotropin
EMS: External Masculinisation Score
