# Correction to: One hundred twelve cases of 46, XY DSD patients after initial gender assignment: a short-term survey of gender role and gender dysphoria

**DOI:** 10.1186/s13023-022-02296-8

**Published:** 2022-03-29

**Authors:** Liping Hou, Ming Zhao, Lijun Fan, Bingyan Cao, Jiajia Chen, Yonghua Cui, Michel Polak, Chunxiu Gong

**Affiliations:** 1 Department of Endocrinology, Genetics and Metabolism, Beijing Children’s Hospital, Capital Medical University, National Center for Children’s Health, Beijing, 100045 China; 2 Department of Endocrinology, Shenzhen Children’s Hospital, Shenzhen, 518038 China; 3 Department of Psychiatry, Beijing Children’s Hospital, Capital Medical University, National Center for Children’s Health, Beijing, 100045 China; 4 Pediatric Endocrinology Diabetology and Gynaecology, Hôpital Universitaire Necker Enfants-Malades, AP-HP, Paris, France

## Correction to: Orphanet Journal of Rare Diseases (2021) 16:416 10.1186/s13023-021-02039-1

Following the publication of the original article [1], it was brought to our attention that the first author is affiliated with the below two institutions:

1. Department of Endocrinology, Genetics and Metabolism, Beijing Children’s Hospital, Capital Medical University, National Center for Children’s Health, Beijing 100,045, China.

2. Department of Endocrinology, Shenzhen Children’s Hospital, Shenzhen 518,038, China.

This is shown in the "Author details" section of this Correction as well as the original article which has now been revised.

